# Combining ability and gene action controlling rust resistance in groundnut (*Arachis hypogaea* L.)

**DOI:** 10.1038/s41598-021-96079-z

**Published:** 2021-08-13

**Authors:** Happy Daudi, Hussein Shimelis, Isack Mathew, Abhishek Rathore, Chris O. Ojiewo

**Affiliations:** 1 African Centre for Crop Improvement, School of Agricultural, Earth and Environmental Sciences, College of Agriculture, Engineering and Science, University of KwaZulu-Natal, Pietermaritzburg, South Africa; 2 Tanzania Agricultural Research Institute-Naliendele, P.O. Box 509, Mtwara, Tanzania; 3 International Crops Research Institute for the Semi-Arid Tropics, Hyderabad, India; 4 International Crops Research Institute for the Semi-Arid Tropics, Nairobi, Kenya

**Keywords:** Genetics, Plant sciences

## Abstract

Groundnut rust caused by *Puccinia arachidis* Speg. is a major cause of yield and quality losses in groundnut (*Arachis hypogaea* L.) in the warm-humid tropics including Tanzania. Breeding and deployment of rust resistant cultivars with farmer-preferred attributes will bolster groundnut production and productivity. The objective of this study was to determine the combining ability effects and gene action controlling rust resistance in groundnut genotypes for breeding. Twelve selected and complementary parental lines were crossed in a diallel design, to develop F_1_ progenies, which were advanced to the F_2_ for individual plant selection. Thirty-three successful partial crosses and the 12 parents were field evaluated using a 5 × 9 alpha lattice designs with two replications over two seasons in Tanzania. The tested genotypes exhibited significant (P < 0.05) variation for rust resistance, yield and yield-related traits. There existed significant (P < 0.05) difference on the general combining ability (GCA) effect of parents and the specific combining ability (SCA) effect of progeny for the assessed traits indicating that both additive and non-additive gene effects conditioned trait inheritance. The Bakers’ ratios indicated that the non-additive gene effects predominantly controlling rust resistance and yield components. This suggested that transgressive segregants could be selected for improved rust resistance and yield gains in the advanced pure line generations. Genotypes ICGV-SM 05570 and ICGV-SM 15567 were the best general combiners for rust resistance and grain yield. The crosses ICGV-SM 16589 × Narinut and ICGV-SM 15557 × ICGV-SM 15559 were identified as the best specific combiners for rust resistance with moderate yield levels and medium maturity. Genotypes with desirable GCA or SCA effects were selected for further breeding.

## Introduction

Groundnut (*Arachis hypogaea* Speg., AABB, 2n = 4x = 40) is cultivated in more than 100 countries in tropical, subtropical and warm temperate regions globally^1^. World groundnut production is estimated at approximately 45.95 million tons of shelled grain per year which is mainly used for oil^2^. Groundnut production in Tanzania is estimated at 0.9 million tonnes per year with an average productivity of less than one tonne per hectare^2^. Despite its importance, groundnut production and productivity are challenged by a number of biotic and abiotic stress factors. Among biotic stresses, groundnut rust disease caused by *Puccinia arachidis* Speg. is a major constraint to groundnut production in the hot humid tropics causing yield losses reaching up to 57%^3^. Reportedly, about 48.3% of groundnut farmers in the hot and humid production environments in Tanzania indicated groundnut rust as the major constraint to high yield and quality^4^.

Groundnut rust causes early pod senescence, reduced seed size, and low seed oil content^3^ reducing the economic value of the crop. Yield losses of up to 70% can be incurred when rust and late leaf spot diseases occur simultaneously^5,6^. Late leaf spot causes leaf senescence significantly reducing the photosynthetic efficiency and leading to yield and quality losses^7^.

Both groundnut rust and late leaf spot diseases can be controlled through a combination of methods such as cultural practices, chemical fungicides, biological control agents and host-plant resistance. Each method has its own merits and demerits when applied in isolation. Host plant resistance is potentially the most economically viable, technically feasible, environmentally friendly, and socially acceptable disease management strategy for groundnut rust integrated disease control^8^. In sub-Sharan Africa host-plant resistance is not widely used as the main rust control strategy due to a lack of varieties with durable disease resistance and enhanced yields. Hence breeding for groundnut rust resistance is the principal consideration to develop better performing varieties with rust resistance and improved productivity. Successful development of improved varieties depends on the genetic variability present in a breeding population and selection of farmer- and market-preferred parents with good combining ability for rust resistance and agronomic traits.

Knowledge on the gene action conditioning economic traits is a prerequisite for breeding resistant groundnut cultivars^9–12^. Evaluating the combining ability of candidate lines is important to identify superior and good combiner parents and progenies, to deduce the type of gene action conditioning trait inheritance and to discern suitable selection methods^13,14^ defined combining ability effects into general combining ability (GCA) of parents and the specific combining ability (SCA) of progeny. The GCA and SCA effects are associated with additive and non-additive gene action, respectively^15^. Both GCA and SCA effects have been reported in foliar disease resistance breeding programs including groundnut using various mating designs^10,16,17^.

The diallel mating design is the most commonly used method to estimate GCA and SCA effects^18^. It is a more appropriate design for self-pollinated species where the success rate for generating crosses is often low such as in groundnut and soybean^19^. It has been used in genetic analysis of traits of various legume crop species such as cowpea^20–22^, soyabean^23,24^ and chickpea^25–27^. To initiate groundnut pre-breeding for rust resistance and farmer-preferred agronomic traits, genetically diverse collections were characterised using agronomic traits and polymorphic simple sequence repeat (SSR) markers. This enabled selection of potential and complementary parents for strategic breeding^28^. The combining ability effects of the selected parents and their progenies should be assessed to develop new breeding populations adapted to Tanzania. Therefore, the objectives of this study were to determine the combining ability effects and gene action controlling rust resistance in selected groundnut genotypes to develop breeding populations.

## Materials and methods

### Description of the study environment

The study was conducted at Tanzania Agriculture Research Institute (TARI), Naliendele Agricultural Research Centre. TARI-Naliendele (10.3539° S, 40.1682° E) is situated at an altitude of 135 m above sea level. The mean monthly temperatures range between 24.3 °C in July and 27 °C in December while the mean annual total rainfall is between 820 and 1245 mm with a unimodal rainfall distribution. A dry spell of one to two weeks often occurs at the end of January or at the beginning of February. The soils at TARI-Naliendele are described as sandy loam with pH of 4.5. The prevailing temperatures and rainfall conditions of the test sites during the experiments are summarised in Table 1.

**Table 1:** Total monthly rainfall and mean maximum and minimum temperature of the test site during 2019 and 2020.

Year	2019			2020			
Month	Total rainfall (mm)	Mean maximum temperature (°C)	Mean minimum temperature (°C)	Month	Total rainfall (mm)	Mean maximum temperature (°C)	Mean minimum temperature (°C)
September	1.0	32.0	21.0	January	289.8	30.8	24.7
October	20.2	32.2	22.8	February	198.4	31.5	24.4
November	55.7	32.2	24.1	March	300.7	32.0	24.1
December	229.9	31.6	24.2	April	102.7	32.3	23.9

### Plant materials

The study used 12 parents selected from preliminary evaluation trials based on rust resistance, agronomic performance and SSR markers^28^. The lines consisted of accessions, a landrace and two released varieties (Table 2). Accessions were selected based on low severity for rust disease or better yield responses (Table 2). A released variety Pendo was included because it is susceptible to rust disease and popular among local farmers in Tanzania. The tested lines included Virginia and Spanish botanical groups (Table 2). The Spanish type have erect growth type and set flowers on their main axis with small capsule^29,30^. The Virginia type have creeping growth type, highly branched main stem with large capsule^29,30^. The selected parents showed varied seed colour and size to cater farmer- and market-preferences (Table 2). The use of plants in the present study complies with international, and or institutional national guidelines and relevant regulations. All required approvals were obtained for the study.

**Table 2:** Description of groundnut parents used in the crosses.

Genotype	Botanical group	Origin	Seed coat colour	Yield (kg ha^−1^)	Rust reaction
ICGV-SM 06737	Spanish	ICRISAT-Malawi	Red	562.50	R
ICGV-SM 05570	Virginia	ICRISAT-Malawi	Red	503.13	R
ICGV-SM 15524	Spanish	ICRISAT-Malawi	Tan	370.63	R
ICGV-SM 15567	Spanish	ICRISAT-Malawi	Tan	543.13	R
ICG 12725	Spanish	ICRISAT-Malawi	Red	308.16	R
ICGV-SM 15559	Spanish	ICRISAT-Malawi	Tan	734.38	R
ICGV-SM 15557	Spanish	ICRISAT-Malawi	Tan	653.13	MR
ICGV-SM 16589	Spanish	ICRISAT-Malawi	Tan	810	MR
ICGV-SM 16601	Spanish	ICRISAT-Malawi	Tan	756.07	MR
Narinut	Virginia	Naliendele/released variety	Tan	202.74	R
Pendo	Spanish	Naliendele/released variety	Tan	753.57	MR
Kanyomwa	Virginia	Landrace	Tan	420.00	R

*ICRISAT* International Crop Research Institute for the Semi-Arid Tropics, *R* resistant, *MR* moderately resistant.

### Crosses and mating design

Crosses were performed using a full diallel mating design involving the 12 lines according to the scheme shown in Table 3. Crossing blocks were established in a greenhouse during the off-season in September 2018. The 12 parents were stagger-planted with a 2-weeks interval to synchronize flowering and pollen supply. Hand emasculation and pollination of the flowers were carried out following the procedure described by^31,32^. Crosses were made during August–October 2018. Emasculation was done between 14:00 and 16:00 h when the hypanthium was sufficiently elongated, the bud was large enough for easy handling during emasculation, and the anthers were not dehisced. Pollination was carried out between 06:30 and 08:00 h the following day. Each cross was labelled appropriately using white tags. A total of 132 cross combinations were expected from the full diallel, however, only 33 crosses had enough seed set (100–200 seeds per cross) for genetic analysis. The F_1_ seed of all successful crosses was planted after three weeks for seed bulking and genetic analysis in the F_2_.

**Table 3:** A 12 × 12 diallel mating scheme in groundnut showing the overall and successful crosses.

Parents	ICG 12725	ICGV-SM 05570	ICGV-SM 06737	ICGV-SM 15524	ICGV-SM 15557	ICGV-SM 15559	ICGV-SM 15567	ICGV-SM 16589	ICGV-SM 16601	Kanyomwa	Narinut	Pendo
ICG 12725	S_1_	ICG 12725 × ICGV-SM 05570	(1) ICG 12725 × ICGV-SM 06737	ICG 12725 × ICGV-SM 15524	ICG 12725 × ICGV-SM 15557	ICG 12725 × ICGV-SM 15559	ICG 12725 × ICGV-SM 15567	ICG 12725 × ICGV-SM 16589	ICG 12725 × ICGV-SM 16601	ICG 12725 × Kanyomwa	ICG 12725 × Narinut	(2) ICG 12725 × Pendo
ICGV-SM 05570	ICGV-SM 05570 × ICG 12725	S_2_	(3) ICGV-SM 05570 × ICGV-SM 06737	ICGV-SM 05570 × ICGV-SM 15524	ICGV-SM 05570 × ICGV-SM 15557	ICGV-SM 05570 × ICGV-SM 15559	ICGV-SM 05570 × ICGV-SM 15567	ICGV-SM 05570 × ICGV-SM 16589	ICGV-SM 05570 × ICGV-SM 16601	ICGV-SM 05570 × Kanyomwa	ICGV-SM 05570 × Narinut	(4) ICGV-SM 05570 × Pendo
ICGV-SM 06737	ICGV-SM 06737 × ICG 12725	(5) ICGV-SM 06737 × ICGV -SM 05570	S_3_	(6) ICGV-SM 06707 × ICGV-SM 15524	ICGV-SM 06737 × ICGV-SM 15557	ICGV-SM 06737 × ICGV-SM 15559	ICGV-SM 06737 × ICGV-SM 15567	(7) ICGV-SM 06737 × ICGV-SM 16589	ICGV-SM 06737 × ICGV-SM 16601	ICGV-SM 06737 × Kanyomwa	(8) ICGV-SM 06737 × Narinut	(9) ICGV-SM 06737 × Pendo
ICGV-SM 15524	ICGV-SM 15524 × ICG 12725	(10) ICGV-SM 15524 × ICGV-SM 05570	ICGV-SM 15524 × ICGV-SM 06737	S_4_	ICGV-SM 15524 × ICGV-SM 15557	ICGV-SM 15524 × ICGV-SM 15559	ICGV-SM 15524 × ICGV-SM 15567	ICGV-SM 15524 × ICGV-SM 16589	ICGV-SM 15524 × ICGV-SM 16601	ICGV-SM 15524 × Kanyomwa	(11) ICGV-SM 15524 × Narinut	ICGV-SM 15524 × Pendo
ICGV-SM 15557	ICGV-SM 15557 × ICG 12725	ICGV-SM 15557 × ICGV-SM 05570	ICGV-SM 15557 × ICGV-SM 06737	ICGV-SM 15557 × ICGV-SM 15524	S_5_	(12) ICGV-SM 15557 × ICGV-SM 15559	ICGV-SM 15557 × ICGV-SM 15567	ICGV-SM 15557 × ICGV-SM 16589	ICGV-SM 15557 × ICGV-SM 16601	ICGV-SM 15557 × Kanyomwa	ICGV-SM 15557 × Narinut	ICGV-SM 15557 × Pendo
ICGV-SM 15559	ICGV-SM 15559 × ICG 12725	(13) ICGV-SM 15559 × ICGV-SM 05570	(14) ICGV-SM 15559 × ICGV-SM 06737	ICGV-SM 15559 × ICGV-SM 15524	ICGV-SM 15559 × ICGV-SM 15557	S_6_	(15) ICGV-SM 15559 × ICGV-SM 15567	ICGV-SM 15559 × ICGV-SM 16589	ICGV-SM 15559 × ICGV-SM 16601	ICGV-SM 15559 × Kanyomwa	ICGV-SM 15559 × Narinut	ICGV-SM 15559 × Pendo
ICGV-SM 15567	ICGV-SM 15567 × ICG 12725	ICGV-SM 15567 × ICGV-SM 05570	ICGV-SM 15567 × ICGV-SM 06737	(15) ICGV-SM 15567 × ICGV-SM 15524	ICGV-SM 15567 × ICGV-SM 15557	ICGV-SM 15567 × ICGV-SM 15559	S_7_	ICGV-SM 15567 × ICGV-SM 16589	ICGV-SM 15567 × ICGV-SM 16601	ICGV-SM 15567 × Kanyomwa	ICGV-SM 15567 × Narinut	ICGV-SM 15567 × Pendo
ICGV-SM 16589	ICGV-SM 16589 × ICG 12725	(16) ICGV-SM 16589 × ICGV-SM 05570	(17) ICGV-SM 16589 × ICGV-SM 06737	ICGV-SM 16589 × ICGV-SM 15524	ICGV-SM 16589 × ICGV-SM 15557	ICGV-SM 16589 × ICGV-SM 15559	(18) ICGV-SM 16589 × ICGV-SM 15557	S_8_	(19) ICGV-SM 16589 × ICGV-SM 16601	ICGV-SM 16589 × Kanyomwa	(20) ICGV-SM 05570 × Narinut	ICGV-SM 16589 × Pendo
ICGV-SM 16601	ICGV-SM 16601 × ICG 12725	ICGV-SM 16601 × ICGV-SM 05570	(21) ICGV-SM 16601 × ICGV-SM 06737	ICGV-SM 16601 × ICGV-SM 15524	ICGV-SM 16601 × ICGV-SM 15557	ICGV-SM 16601 × ICGV-SM 15559	ICGV-SM 16601 × ICGV-SM 15567	ICGV-SM 16601 × ICGV-SM 16589	S_9_	ICGV-SM 16601 × Kanyomwa	(22) ICGV-SM 05570 X Narinut	ICGV-SM 16601 × Pendo
Kanyomwa	Kanyomwa × ICG 12725	(23) Kanyomwa × ICGV-SM 05570	Kanyomwa × ICGV-SM 06737	Kanyomwa × ICGV-SM 15524	Kanyomwa × ICGV-SM 15557	(24) Kanyomwa × ICGV-SM 15559	Kanyomwa × ICGV-SM 15567	Kanyomwa × ICGV-SM 16589	Kanyomwa × ICGV-SM 16601	S_10_	(25) Kanyomwa × Narinut	Kanyomwa × Pendo
Narinut	Narinut × ICG 12725	(26) Narinut × ICGV-SM 05570	(28) Narinut × ICGV-SM 06737	Narinut × ICGV-SM 15524	Narinut × ICGV-SM 15557	Narinut × ICGV-SM 15559	Narinut × ICGV-SM 15567	Narinut × ICGV-SM 16589	Narinut × ICGV-SM 16601	Narinut × Kanyomwa	S_11_	Narinut × Pendo
Pendo	Pendo × ICG 12725	(29) Pendo × ICGV-SM 05570	Pendo × ICGV-SM 06737	(30) Pendo × ICGV-SM 15524	Pendo × ICGV-SM 15557	Pendo × ICGV-SM 15559	Pendo × ICGV-SM 15567	(31) Pendo × ICGV-SM 16589	(32) Pendo × ICGV-SM 16601	Pendo × Kanyomwa	(33) Pendo × Narinut	S_12_

Numbers (1) to (33) denote successful crosses, which were used for genetic analyses.

S_1_ to S_12_ denote selfs.

### Experimental design and trial management

Thirty-three successful progenies and their parents were evaluated in the field using a 5 × 9 alpha lattice designs with two replications at TARI-Naliendele in two seasons (2019 and 2020). The genotypes were evaluated for rust resistance, agronomic performance and yield potential during the off-season in 2019 and the rainy season in 2020. The off-season trial was conducted under irrigation, while the main season trial was done under rainfed condition. Each genotype was planted on two rows of 4 m length, with a spacing of 50 cm between the rows and 10 cm between plants in a row. The plot size for each genotype was 2.0 m^2^. The recommended practices for fertilizer application and weeding for groundnut were followed^33^. This site is a hotspot for rust and late leaf spot diseases. Hence genotypes were evaluated under natural rust and late leaf spot infection and disease development.

### Data collection

Data on yield and yield components were recorded during plant growth and at harvest maturity. Days to flowering (DTF) were recorded by counting the number of days from sowing to the time when 75% of the plants in a plot had emerging flowers. Days to maturity (DTM) were recorded by counting the number of days from planting to maturity. Plant height (PH, expressed in cm) was measured from ten randomly sampled plants in each plot from the soil surface to the tip of main stem. The number of pods per plant (NPP) was recorded as the average number of pods from ten randomly sampled and tagged plants per plot. Pod yield (PDY) was measured by weighing the dried pods from each plot and was recorded in grams per plot. Shelling percentage (SP) for each genotype was calculated from a random sample of pods weighing 200 g, as the proportion of shelled seed weight to the total weight of the unshelled pods. Additionally, 100 seed weight (HSW, expressed in grams) for each genotype was recorded as an average weight of two samples of 100 well-developed whole air-dried kernel per plot. Kernel yield (KY, expressed in t ha^−1^) was estimated as the weight of kernels harvested from a plot.

Rust severity was scored at 85 days after planting (%RI85) and 100 days after planting (%RI100). The severity was scored using a scale of 1 (least affected) to 9 (most affected) following^34^. Plants with no symptoms of infection were assigned a disease score of 1 (for 0% infection) while leaves with 1–5% infection were assigned a score of 2, 6–10% infection (score 3), 11–20% infection (score 4), 21–30% (score 5), 31–40% infection (score 6), 41–60% infection (score 7), 61–80% infection (score 8) and 81–100% infection (score 9)^35^. Plants with a disease score of between 1 and 3, 4 and 6, and 7 and 9 were considered as resistant, moderately resistant and susceptible, respectively^36^. In addition, late leaf spot reaction was assessed as a secondary trait due to the simultaneous occurrence with rust disease. Late leaf spot severity was assessed at 85 days after planting (%LLSI85) and 100 days after planting (%LLSI100) as in rust severity. For data analysis, the score data were converted into percentage severity using^37^.

### Data analyses

#### Estimation of combining abilities

The data were subjected to analysis of variance (ANOVA) using SAS 9.4^38^ and means were separated by Fischers unprotected least significant difference at 5% probability level. General combining ability (GCA) effects of parents and specific combining (SCA) of crosses were computed based on the partial diallel analysis proposed by^39^. The general linear model used was as follows:

$${\mathrm{Y}}_{\mathrm{ijk}}=\upmu + {\mathrm{g}}_{\mathrm{i}} + {\mathrm{g}}_{\mathrm{j}} + {\mathrm{s}}_{\mathrm{ij}}+{r}_{ij}+{b}_{k}+ {\mathrm{e}}_{\mathrm{ijk}}$$

where ${\mathrm{Y}}_{\mathrm{ijk}}$ is the observed measurement for the ${\mathrm{ij}}^{\mathrm{th}}$ cross grown in the ${\mathrm{k}}^{\mathrm{th}}$ replication or environment; $\upmu$ is the population mean; ${\mathrm{g}}_{\mathrm{i}}$, and ${\mathrm{g}}_{\mathrm{j}}$ are the GCA effects; ${\mathrm{s}}_{\mathrm{ij}}$ the SCA effect; r_ij_ is the reciprocal cross effect between i^th^ and j^th^ parents; b_k_ is the effect of the kth block and ${\mathrm{e}}_{\mathrm{ijk}}$ the error term associated with the ${\mathrm{ij}}^{\mathrm{th}}$ cross evaluated in the ${\mathrm{k}}^{\mathrm{th}}$ replication or environment.

The relative importance of GCA and SCA effects was estimated using the GCA-SCA prediction ratio proposed by^40^ as follows:

$$\frac{{2{\sigma }^{2}}_{GCA}}{{2{\sigma }^{2}}_{GCA}+{{\sigma }^{2}}_{SCA}}$$

where: ${{\sigma }^{2}}_{GCA}$ and ${{\sigma }^{2}}_{SCA}$ are estimated variance components for GCA and SCA effects, respectively.

A trait whose Bakers’ ratio is close to 1.00 indicates that the GCA effects were more important in conditioning the heritability of that trait, whereas a ratio that is close to zero would indicates that SCA effects would be more important in controlling trait heritability.

## Results

### Genetic variation and mean yield and yield component response of parents and progeny

The ANOVA revealed that the genotype × season interaction effects had significant (P < 0.001) impact on DTF and NPP (Table 4). There was wide genotypic variation for all the assessed traits except SP. The traits DTF, NPP, DTM and SP exhibited significant (P < 0.05) seasonal variability (Table 4).

**Table 4:** Analysis of variance showing F-statistic values for four disease parameters and eight agronomic traits of 45 groundnut genotypes evaluated in two seasons in Tanzania.

Source of variation	Disease parameters					Agronomic traits							
	DF	%RI85	%RI100	%LLS85	%LLS100	DTF	PH	NPP	DTM	PDY	HSW	SP	KY
Replication	1	0.29	2.40	0.29	0.09	5.61*	0.48	1.89	0.01	0.22	0.75	0.38	0.13
Block (Replication)^$^	8	2.70	3.40	0.10	3.30	0.10	0.50	0.80	0.50	8.20**	3.50	0.10	7.20**
Genotype (G)	44	5.46***	5.39***	4.09***	4.26***	6.16***	62.26***	2.68***	3.66***	6.86***	5.61***	1.27	5.72***
Season (S)	1	0.48	0.41	0.01	1.12	5.14*	1.36	150.05***	11.39**	0.01	0.01	9.48**	0.70
G × S	44	0.22	0.33	0.35	0.81	2.15***	0.62	1.98***	0.24	0.26	0.99	0.54	0.32
Residual		0.06	0.07	0.03	0.05	7.15	6.12	0.95	32.95	333,741	65.64	0.02	164,336

*DF* degrees of freedom, *%RI85* percentage rust infection at 85 days after planting, *%RI100* percentage rust score infection at 100 days after planting, *%LLSI 85* percentage late leaf spot infection at 85 days after planting, *%LLSI 100* percentage late leaf spot infection at 100 days after planting, *DTF* days to flowering, *PH* plant height, *NPP* number of pods per plant, *DTM* days to maturity, *PDY* pod yield, *HSW* hundred seed weight, *SP* shelling percent, *KY* kernel yield.

^$^Indicates chi-square statistic.

*,** and *** represent significant differences at 0.05, 0.01 and 0.001 probability levels, respectively.

Days to flowering varied from 31 days (for cross ICGV-SM06737 × Pendo) to 45 days (cross ICGV-SM16589 × ICGV-SM05570) (Table 5). The earliest maturing genotypes included crosses ICG12725 × Pendo, ICGV-SM06737 × ICGV-SM16589, ICGV-SM15524 × Narinut, and Pendo × ICGV-SM16601, which matured in 99 days. Cross Narinut × ICGV-SM 05570 had the highest number of pods per plant (32 pods plant^−1^). The highest HSW was recorded for the cross ICG 12725 × ICGV-SM 06737 (46.35 g/100 seed). The highest pod yield of 4712.82 kg ha^−1^ was attained by the cross ICGV-SM 15524 × ICGV-SM 05570. The crosses that had better yield response than their mid parents included ICGV-SM 15524 × ICGV-SM 05570 (1548.72 kg ha^−1^), Narinut × ICGV-SM 05570 (957.23 kg ha^−1^), Pendo × ICGV-SM 15524 (908.36 kg ha^−1^), and Pendo × ICGV-SM 16601 (864.37 kg ha^−1^).

**Table 5:** Mean values of four disease parameters and eight agronomic traits of 12 parental genotypes of groundnut and their 33 F_2_ families evaluated in two seasons in Tanzania.

Entry	Disease parameters				Agronomic traits							
	%RI85	%RI100	%LLSI85	%LLSI100	DTF	PH	NPP	DTM	PDY	HSW	SP	KY
**Parents**												
ICG12725	21.05	35.54	8.75	11.01	37	20.30	14	113	890.10	30.51	64.74	587.23
ICGV-SM05570	6.58	11.52	2.50	6.05	38	23.25	13	110	1146.10	28.01	63.56	725.30
ICGV-SM06737	3.84	8.14	3.92	12.38	40	25.16	13	112	1044.43	26.73	58.41	640.33
ICGV-SM15524	12.70	18.63	7.43	17.55	38	22.64	13	107	975.67	24.99	64.77	660.45
ICGV-SM15557	4.92	14.01	2.26	4.75	38	24.18	9	99	489.41	19.06	56.15	268.56
ICGV-SM15559	5.97	10.69	1.82	6.66	38	22.47	11	110	1053.29	22.39	56.02	595.72
ICGV-SM15567	0.71	3.95	22.67	34.96	38	24.78	15	110	1724.95	21.77	58.19	970.97
ICGV-SM16589	15.51	26.71	6.40	14.98	38	29.87	11	110	779.79	23.79	54.20	436.63
ICGV-SM16601	14.79	29.22	6.24	16.34	39	23.60	10	108	887.70	28.60	64.13	587.70
Kanyomwa	11.30	18.54	0.61	4.00	39	27.31	16	109	832.47	25.51	56.12	491.22
Narinut	16.96	28.12	4.36	14.31	38	20.44	12	110	768.35	22.92	57.25	441.51
Pendo	28.85	39.61	15.65	28.38	37	23.74	8	107	841.04	27.31	62.72	530.49
**Crosses**												
ICG12725 × ICGV-SM06737	1.43	2.60	2.31	4.10	35	37.15	12	113	1639.31	46.35	58.31	943.05
ICG12725 × Pendo	61.26	63.81	30.06	57.55	35	43.30	19	99	1294.64	26.49	63.49	839.17
ICGV-SM05570 × ICGV-SM06737	0.00	0.00	1.31	1.93	36	39.40	22	110	1666.27	20.95	60.58	1004.14
ICGV-SM05570 × Pendo	4.70	10.38	0.00	0.00	34	28.70	10	113	1581.73	34.60	57.94	813.71
ICGV-SM06737 × ICGV-SM15524	0.36	0.36	3.76	7.54	36	28.55	24	107	1689.11	10.42	59.89	984.49
ICGV-SM06737 × ICGV-SM16589	36.82	55.17	21.58	40.88	35	39.33	14	99	1560.88	21.54	58.86	892.50
ICGV-SM06737 × Narinut	32.47	39.98	1.99	15.30	36	32.23	15	114	1280.38	40.16	62.13	776.83
ICGV-SM06737 × Pendo	15.72	32.47	7.31	11.00	31	26.50	12	106	1502.81	5.35	58.10	825.30
ICGV-SM15524 × Narinut	43.75	57.51	7.55	20.03	33	45.95	10	99	656.53	27.20	52.85	336.97
ICGV-SM15557 × ICGV-SM15559	4.67	6.83	2.00	6.55	42	25.35	19	114	1510.60	27.78	61.85	930.13
ICGV-SM 15559 × ICFV-SM 15567	23.2	17.8	21.30	20.2	38	42.13	9	114	850	22.45	54.05	459.43
ICGV-SM16589 × ICGV-SM16601	55.01	75.09	19.46	33.46	35	41.06	14	110	1318.93	26.60	58.24	774.16
ICGV-SM16589 × Narinut	1.93	11.17	1.36	9.25	36	26.30	13	113	1461.05	19.67	50.02	740.25
ICGV-SM16601 × Narinut	7.30	17.43	3.74	8.32	35	22.03	18	100	1610.67	35.26	54.37	958.20
Kanyomwa × Narinut	4.67	4.67	0.00	2.65	39	36.29	14	114	1321.45	30.04	53.98	744.00
ICGV-SM06737 × ICGV-SM05570	1.32	8.32	0.35	1.32	35	40.33	15	114	1212.34	39.75	64.38	789.30
ICGV-SM15524 × ICGV-SM05570	0.00	0.00	1.27	2.88	37	41.28	30	114	4712.82	37.17	63.99	3018.25
ICGV-SM15559 × ICGV-SM05570	4.67	9.87	3.99	8.99	35	39.78	21	113	1818.15	31.98	54.49	1033.01
ICGV-SM15559 × ICGV-SM06737	1.32	1.32	0.00	0.00	45	19.15	6	114	1740.56	19.11	52.88	914.63
ICGV-SM15567 × ICGV-SM15524	0.71	0.00	0.01	0.00	35	29.45	15	113	1337.45	30.77	66.82	884.32
ICGV-SM16589 × ICGV-SM05570	0.36	1.32	0.01	0.00	45	32.28	23	113	1565.74	21.91	71.22	1085.73
ICGV-SM16589 × ICGV-SM06737	0.36	1.32	6.01	8.32	39	49.98	18	114	1099.27	3.55	43.06	496.12
ICGV-SM16589 × ICGV-SM15557	42.49	57.51	0.95	13.33	36	33.19	14	113	1106.97	42.69	59.52	641.75
ICGV-SM16601 × ICGV-SM06737	0.00	0.00	0.00	3.29	38	27.65	13	113	1837.97	36.68	53.58	985.58
Kanyomwa × ICGV-SM05570	3.29	5.39	4.60	11.17	33	31.63	18	106	1663.36	22.20	53.40	1003.44
Kanyomwa × ICGV-SM15559	1.32	5.00	0.36	1.32	36	21.20	12	114	2240.61	27.82	64.95	1464.50
Narinut × ICGV-SM05570	0.00	0.00	0.35	1.32	37	27.08	32	114	2452.21	41.29	63.41	1576.17
Narinut × ICGV-SM06737	6.83	9.06	4.75	5.39	36	36.43	20	110	1131.60	18.65	55.62	635.25
Pendo × ICGV-SM05570	0.00	0.00	0.01	0.36	38	28.20	18	113	1260.74	28.12	47.88	600.81
Pendo × ICGV-SM15524	1.32	13.50	17.38	22.45	36	43.13	17	114	2082.72	31.39	68.95	1440.49
Pendo × ICGV-SM16589	46.18	55.01	27.61	37.42	33	39.38	19	100	1470.73	25.37	59.53	853.52
Pendo × ICGV-SM16601	8.60	22.45	1.34	17.13	44	21.20	14	99	1686.72	16.34	63.89	1106.66
Pendo × Narinut	24.29	32.05	14.08	27.98	35	27.75	16	113	1173.45	44.91	60.77	696.97
Mean	12.64	19.19	6.09	12.56	37	30.66	15	110	1434.57	27.13	58.98	857.40
CV (%)	81.68	69.79	89.64	69.85	7.24	8.07	25.07	5.24	40.27	29.86	14.25	47.28
LSD (5%)	0.30	0.33	0.22	0.27	3.30	2.92	1.20	7.19	730.49	10.24	**-**	512.20

*%RI85* percentage rust infection at 85 days after planting, *%RI100* percentage rust score infection at 100 days after planting, *%LLSI 85* Percentage late leaf spot infection at 85 days after planting, *%LLSI 100* percentage late leaf spot infection at 100 days after planting, *DTF* days to flowering, *PH* plant height, *NPP* number of pods per plant, *DTM* days to maturity, *PDY* pod yield, *HSW* hundred seed weight, *SP* shelling percent, *KY* kernel yield, *LSD* least significant difference, *CV* coefficient of variation.

### Combining ability of groundnut genotypes for rust and leaf spot resistance and agronomic traits

The mean squares for GCA, SCA and reciprocal variance for disease parameters and eight agronomic traits are presented in Table 6. The GCA, SCA and reciprocal effects were highly significant for all the traits except for NPP whose GCA variance was not significant (Table 6). Seasonal effects significantly affected the GCA, SCA and reciprocal variances for NPP and HSW only (Table 6).

**Table 6:** Analysis of variance showing F-statistic for combining ability effects for four disease parameters and eight agronomic traits of 45 groundnut genotypes evaluated in two seasons in Tanzania.

Source of variation	DF	Disease parameters				Agronomic parameters							
		%RI85	%RI100	%LLS85	%LLS100	DTF	PH	NPP	DTM	PDY	HSW	SP	KY
GCA	11	7.21***	7.79***	6.11***	6.51***	3.45***	91.88***	0.93	2.34**	4.01***	8.25***	0.84	3.45***
SCA	19	4.14***	3.51***	2.81***	2.86***	2.66***	88.75***	2.0**	4.38***	3.74***	7.60***	0.71	2.66***
GCA*ENV	11	0.14	0.25	0.42	0.84	0.29	0.28	2.22*	0.22	0.32	1.49	0.51	0.29
SCA*ENV	19	0.11	0.14	0.32	0.66	0.24	0.66	1.60*	0.21	0.19	2.01**	0.50	0.24
REC	14	2.65**	3.64***	2.78***	3.63***	9.33***	27.25***	2.58**	2.37**	10.84***	6.86***	1.38	9.33***
REC*ENV	14	0.14	0.28	0.27	0.90	0.25	1.02	2.26**	0.16	0.10	1.23	0.34	0.25

*DF* degrees of freedom, *%RI85* percentage rust infection at 85 days after planting, *%RI100* percentage rust score infection at 100 days after planting, *%LLSI 85* Percentage late leaf spot infection at 85 days after planting, *%LLSI 100* percentage late leaf spot infection at 100 days after planting, *DTF* days to flowering; *PH* plant height, *NPP* number of pods per plant, *DTM* days to maturity, *PDY* pod yield, *HSW* hundred seed weight, *SP* shelling percent, *KY* kernel yield, *GCA* general combining ability, *SCA* specific combining ability, *ENV* environment/season; *REC* reciprocal, GCA*ENV general combining ability by environment/season interaction, SCA*ENV specific combining ability by environment/season interaction, REC*ENV reciprocal by environment/season interaction.

*,** and *** represent significant differences at 0.05, 0.01 and 0.001 probability levels, respectively.

### General combining ability effects

The GCA estimates varied among the 12 parental genotypes for the agronomic traits and disease parameters (Table 7). The best combiners for %RI85 and %RI100 were ICGV-SM 05570 and ICGV-SM 15567, which had negative and desirable GCA effects of − 0.15 and − 0.14, respectively. In addition, ICGV-SM 05570 exhibited negative and desirable GCA effects for %LLSI100. Desirable GCA effects for DTF and DTM were exhibited by genotypes ICGV-SM 15524, and ICGV-SM 16601, respectively. There was only one parental line, Kanyomwa, which exhibited desirable GCA effect for pod and kernel yield. Genotype Kanyomwa was the best general combiner for kernel and pod yield with GCA effects of 760.61 and 1007.47, respectively, while Narinut 15 had significant negative effects for both (Table 7).

**Table 7:** General combining ability effects with mean squares and significant tests for four disease parameters and eight agronomic traits of 12 parental genotypes evaluated in two seasons in Tanzania.

Parents	Disease parameters				Agronomic traits							
	%RI85	%RI100	%LLSI85	%LLSI100	DTF	PH	NPP	DTM	PDY	HSW	SP	KY
ICG 12725	0.07	0.06	0.14**	0.10	1.70*	− 1.79**	− 0.14	− 0.56	− 205.07	− 3.23	0.04	− 68.54
ICGV-SM 05570	− 0.15**	− 0.16*	− 0.06	− 0.13*	2.25**	− 4.09	− 0.10	1.75	− 159.42	− 6.64**	0.02	− 78.59
ICGV-SM 06737	− 0.11	− 0.10	0.01	− 0.02	1.19	− 3.77	− 0.08	1.27	110.21	− 13.06	0.04	107.40
ICGV-SM 15524	− 0.08	− 0.21	− 0.05	− 0.08	− 6.05*	29.44	1.14	− 6.90	− 398.30	21.52*	− 0.21	− 511.29
ICGV-SM 15557	0.26*	0.29*	− 0.22*	− 0.06	− 0.20	− 3.67**	− 0.14	4.65	− 174.42	18.04	0.05	− 60.60
ICGV-SM 15559	− 0.05	− 0.06	− 0.02	− 0.03	1.49**	− 5.57	− 0.34*	0.43	− 179.85*	− 6.52	− 0.005	− 108.17
ICGV-SM 15567	− 0.14*	− 0.14*	0.16**	0.14**	1.15	− 4.42	− 0.01	0.48	155.98	− 6.83**	0.01	79.45
ICGV-SM 16589	0.12*	0.16**	0.14**	0.14**	0.58	3.25	− 0.06	− 0.65	− 131.60	− 10.79	− 0.03	− 109.72
ICGV-SM 16601	0.02	0.09	0.04	0.06	3.28	− 6.82	− 0.17	− 3.74*	2.17	− 8.19	0.04	74.68
Kanyomwa	− 0.17	− 0.15	− 0.10	− 0.18	− 0.62	− 6.84	− 0.16	3.94	1007.47**	− 1.09	0.09	760.61**
Narinut	0.04	0.06	0.02	0.03	1.61**	− 6.59	− 0.23	0.35	− 322.32**	− 6.26	0.001	− 185.28**
Pendo	0.19	0.16	− 0.05	0.01	− 6.38	10.87	0.31	− 1.02	295.15	23.04	− 0.04	100.04

*%RI85* percentage rust infection at 85 days after planting, *%RI100* percentage rust score infection at 100 days after planting, *%LLSI 85* Percentage late leaf spot infection at 85 days after planting, *%LLSI 100* percentage late leaf spot infection at 100 days after planting, *DTF* days to flowering, *PH* plant height, *NPP* number of pods per plant, *DTM* days to maturity, *PDY* pod yield; *HSW* hundred seed weight, *SP* shelling percent, *KY* kernel yield.

*,** and *** represent significant differences at 0.05, 0.01 and 0.001 probability levels, respectively.

### Specific combining ability effects

The SCA effects of the 33 crosses for the twelve characters showed a wide variation (Table 8). Good specific combiners for rust infection were ICGV-SM 15557 × ICGV-SM 15559, ICGV-SM 16589 × Narinut and ICG 12725 × ICGV-SM 06737, which exhibited negative SCA effects for %RI85 or %RI100 (Table 8). Crosses Pendo × ICGV-SM 05570 exhibited desirable negative and significant (P ≤ 0.05) SCA effects for DTF, while Kanyomwa × ICGV-SM 05570 had positive and significant SCA effect for DTF. Crosses that exhibited negative and significant (P ≤ 0.05) SCA effects for DTM included ICG 12725 × Pendo and ICGV-SM 16589 × ICGV-SM 06737. None of the families exhibited positive and significant (P ≤ 0.01) effect for KY, although crosses ICGV-SM 06737 × ICGV-SM 15524 and Kanyomwa × ICGV-SM 05570 showed high and positive SCA effect for KY.

**Table 8:** Specific combining ability effects showing mean squares and significant tests for four disease parameters and eight agronomic traits of 33 F_2_ families evaluated in two seasons in Tanzania.

Crosses	Disease parameters				Agronomic traits							
	%RI85	%RI100	%LLSI85	%LLSI100	DTF	PH	NPP	DTM	PDY	HSW	SP	KY
ICG 12725 × Pendo	0.25	0.21	0.32**	0.41**	4.77**	0.62	0.22	− 8.33*	− 208.43	− 28.75	0.07	− 4.39
Narinut × ICGV-SM05570	0.22	0.31*	0.07	0.09	1.95	− 4.14**	− 2.05**	− 2.44	− 1520.96	− 8.75	− 0.04	− 1027.97
ICGV-SM 15524 × ICGV-SM 05570	0.06	0.02	− 0.04	− 0.10	− 5.12	17.68	− 0.49	− 9.69	− 3857.55	13.15	− 0.26	− 2796.06
Pendo × ICGV-SM 05570	0.15	0.22*	− 0.01	0.01	− 2.25*	0.25	− 0.49	− 0.05	160.50	3.24	0.05	106.45
ICGV-SM 16589 × ICGV-SM 05570	0.25	0.31*	0.25*	0.33*	− 7.28	0.50	− 0.99	− 2.78	− 443.76	− 3.91	− 0.15*	− 461.97
Kanyomwa × ICGV-SM 05570	− 0.17	− 0.11	− 0.20	− 0.31	3.50	− 8.95	− 0.54	8.95*	597.68	5.51	0.15	490.64
ICGV-SM 06737 × ICGV-SM 05570	− 0.16	− 0.16	0.03	0.01	0.12	− 0.46	0.41	− 1.69	226.97	− 9.40**	− 0.02	107.42
Narinut × ICGV-SM 06737	0.17*	0.19*	− 0.04	0.09	0.16	− 2.1*	− 0.25	1.97	74.39	10.75**	0.03	70.79
Kanyomwa × ICGV-SM 15559	0.00	0.00	0.00	0.00	0.00	0.00	0.00	0.00	0.00	0.00	0.00	0.00
ICGV-SM 15559 × ICGV-SM 05570	− 0.08	− 0.11	− 0.10	− 0.15	4.41**	− 15.83	− 1.14*	− 1.98	− 744.43*	− 9.71*	0.04	− 407.71
ICGV-SM 15557 × ICGV-SM 15559	− 0.36	− 0.43*	0.21	0.03	5.59**	0.98	0.86	− 0.65	451.89	− 19.17**	0.003	286.84
ICG 12725 × ICGV-SM 06737	0.21	− 0.26*	− 0.17*	− 0.21*	− 2.83*	9.10	− 0.25	3.20	321.18	27.21	− 0.07	92.13
ICGV-SM 06737 × ICGV-SM 15524	− 0.06	− 0.02	0.06	0.09	5.97*	− 30.73	− 0.11	3.13	564.21	− 33.47**	0.20	576.32
ICGV-SM 16589 × ICGV-SM 06737	0.32**	0.38**	0.12	0.21**	− 2.25*	− 5.33	− 0.22	− 7.32**	230.81	8.99**	0.08	198.19
ICGV-SM 16601 × ICGV-SM 06737	0.21	0.40*	0.22*	0.17	2.14	− 4.63**	0.13	− 6.47	− 312.61	− 22.50	0.11	8.57
ICGV-SM 15559 × ICGV-SM 06737	0.07	0.19	0.16	0.26*	− 7.39	5.12**	1.01	− 2.77	− 397.21	− 3.26	0.07	− 103.35
ICGV-SM 15524 × Narinut	0.38	0.51	0.13	0.14	1.77	− 10.51	− 1.60	− 3.61	− 35.85	− 23.50**	0.17	221.47
ICGV-SM 05570 × ICGV-SM 06737	0.01	0.00	− 0.04	− 0.04	− 3.27**	14.11	0.52	− 0.17	75.53	14.62	− 0.004	55.84
ICGV-SM 15567 × ICGV-SM 15524	0.13	0.05	0.28	0.40	− 4.71	29.18	1.15	10.63	− 166.78	19.36	− 0.31	− 504.09
Kanyomwa × Narinut	0.02	− 0.04	− 0.10	0.03	2.35	11.74	0.25	0.38	− 776.70	1.96	− 0.12	− 643.4
ICGV-SM 06737 × Narinut	0.016	0.10	− 0.02	− 0.02	− 2.33*	11.07	0.56	0.65	5.11	13.29**	− 0.02	− 28.14
Pendo × Narinut	0.00	0.00	0.00	0.00	0.00	0.00	0.00	0.00	0.00	0.00	0.00	0.00
Pendo × ICGV-SM 15524	− 0.21	− 0.90	− 0.74	− 0.77	− 35.86*	141.88	5.56	− 23.72	− 1660.53	159.07**	− 1.26	− 2708.43
ICGV-SM 15559 × ICGV-SM 15567	0.15	0.10	0.21	0.17	− 1.19	12.05	1.35	− 7.15	64.17	2.15	− 0.05	78.25
ICGV-SM 05570 × Pendo	0.25	− 0.25	− 0.06	− 0.16	4.99**	− 11.94	− 0.43	3.39	− 127.48	− 20.47	− 0.02	− 126.25
ICGV-SM 16589 × Narinut	− 0.36**	− 0.33*	− 0.22**	− 0.19	− 1.16	− 3.98**	0.03	4.35	501.97	1.28	− 0.05	223.18
ICGV-SM 16601 × Narinut	− 0.12	− 0.15	− 0.04	− 0.12	− 5.01**	1.82	0.71	− 6.25*	517.83	14.27**	− 0.07	256.73
ICGV-SM 16589 × ICGV-SM 15557	0.00	0.00	0.00	0.00	0.00	0.00	0.00	0.00	0.00	0.00	0.00	0.00
Pendo × ICGV-SM 16589	0.00	0.00	0.00	0.00	0.00	0.00	0.00	0.00	0.00	0.00	0.00	0.00
ICGV-SM 06737 × ICGV-SM 16589	0.04	0.02	0.04	0.07	− 0.19	11.56	0.20	− 3.35	− 61.53	0.97	− 0.07	− 115.44
ICGV-SM 16589 × ICGV-SM 16601	0.40**	0.42**	0.10	0.12	− 4.01	11.02	0.001	5.08*	35.38	10.14**	− 0.002	− 2.87
Pendo × ICGV-SM 16601	0.00	0.00	0.00	0.00	0.00	0.00	0.00	0.00	0.00	0.00	0.00	0.00
ICGV-SM 06737 × Pendo	− 0.03	0.09	0.14	0.03	0.57	− 14.21	− 0.73	− 3.20	− 315.53	− 40.05	0.02	− 194.19

*%RI85* percentage rust infection at 85 days after planting, *%RI100* percentage rust score infection at 100 days after planting, *%LLSI 85* Percentage late leaf spot infection at 85 days after planting, *%LLSI 100* percentage late leaf spot infection at 100 days after planting, *DTF* days to flowering; *PH* plant height; *NPP* number of pods per plant, *DTM* days to maturity, *PDY* pod yield, *HSW* hundred seed weight, *SP* shelling percent, *KY* kernel yield.

*,** and *** represent significant differences at 0.05, 0.01 and 0.001 probability levels, respectively.

### Gene action

The GCA variances were smaller than the corresponding SCA variances for all assessed traits (Table 9). The Bakers ratios which are based on the GCA to SCA variance were all below 0.50 suggesting the preponderance of non-additive genetic effects. In comparison to agronomic traits, disease-related traits such as %RI100, %LLS185, and %LLSI100 had higher Bakers’ ratios, each with about a value of 0.20. The broad sense heritability of assessed traits ranged between 17.89 and 97.74%. The highest heritability was estimated for plant height at 97.74% (Table 9).

**Table 9:** Variance components for four-disease parameters and eight agronomic traits of 45 groundnut genotypes evaluated in two seasons.

Traits	GCA	SCA	REC	GCA & SCA ratio	Baker’s ratio	H^2^ (%)
**Disease parameters**						
%RI85	0.008	0.092	0.018	0.085	0.145	78.48
%RI100	0.011	0.095	0.039	0.111	0.182	79.89
%LLSI85	0.003	0.029	0.013	0.113	0.184	70.47
%LLSI100	0.005	0.046	0.026	0.117	0.190	68.40
**Agronomic traits**						
DTF	0.559	22.318	11.635	0.025	0.048	65.38
PH	10.081	256.013	37.641	0.039	0.073	97.74
NPP	− 0.005	0.545	0.383	− 0.008	− 0.017	20.26
DTM	1.136	53.976	14.639	0.021	0.040	70.27
PDY	21,812.010	480,854.190	830,693.490	0.045	0.083	83.13
HSW	9.638	220.665	97.726	0.044	0.080	80.46
SP	− 0.000	− 0.002	0.001	0.009	0.018	17.89
KY	8655.410	146,365.650	348,164.330	0.059	0.106	80.53

*%RI85* percentage rust infection at 85 days after planting, *%RI100* percentage rust score infection at 100 days after planting, *%LLSI85* Percentage late leaf spot infection at 85 days after planting; *%LLSI100* percentage late leaf spot infection at 100 days after planting, *DTF* days to flowering, *PH* plant height, *NPP* number of pods per plant, *DTM* days to maturity, *PDY* pod yield, *HSW* hundred seed weight, *SP* shelling percent, *KY* kernel yield, *GCA* general combining ability, *SCA* specific combining ability, *REC* reciprocal, *H*^*2*^ broad sense heritability.

## Discussion

### Genetic variations among parents and progeny

The F_2_ progeny and their parents showed significant (P < 0.05) variation for yield and yield components, suggesting that the test genotypes were useful genetic resources for groundnut improvement. The groundnut genotypes used in this study included divergent parental lines from Virginia and Spanish botanical groups which invariably contributed to the genetic variation observed in the new breeding population. The parental lines have different genetic constitution, agronomic potential and adaptations providing the F_2_ progeny with transgressive segregations. This led to wide genetic variation in the observed performance in the F_2_. The presence of high progeny performance exceeding the parental phenotypic values have been reported in segregating populations^41–43^. Transgressive segregation is useful in crop improvement as one of the mechanisms that contribute to genotype discovery with unique genetic composition and novel adaptations compared to the base population^44^. Crop improvement depends on the availability of genetic variation with stable performance for economic traits^45^. Genotype × season interaction effects were significant for DTF and NPP suggesting that seasonal variability and change in climatic conditions affects the phenotypic expression of the tested groundnut genotypes. Seasonal variability presents challenges for selection as it reduces correlation between genotype and phenotypic expression. Genotypes such as ICGV-SM 15524 × ICGV-SM 05570 and Kanyomwa × ICGV-SM 15559 that consistently perform across seasons and locations will be ideal for selection. Traits whose expression was not significantly affected by seasonal or genotype × seasonal variability will be easier to select and improve. Mekontchou et al.^46^ and Bucheyeki et al.^47^ reported significant genotype, environmental and genotype × environment interaction variations for agronomic traits in groundnuts.

The impact of rust diseases on groundnut production compels breeders to select genotypes that express appreciable levels of rust resistance coupled with high yield potential. Parental lines such as ICGV-SM 15567 and progeny such as ICGV-SM 15524 × ICGV-SM 05570 and Narinut × ICGV-SM 05570 exhibited low rust infection and had high kernel yield response across seasons. Higher mean performance among crosses compared to the parents indicates that there was genetic gain in yield and agronomic performance among the crosses. For groundnut rust resistance breeding, the following families were selected: ICGV-SM 15524 × ICGV-SM 05570, Pendo × ICGV-SM 05570, ICGV-SM 16601 × ICGV-SM 06737 and ICGV-SM 05570 × ICGV-SM 06737. These families exhibited low rust severity than their corresponding parents. This suggests that rust resistance was achieved through gene recombination hence desirable transgressive segregants can be selected in successive generations. For instance, Pendo is known to be susceptible to rust but its progeny, i.e., Pendo × ICGV-SM 05570 was among the crosses with desirable SCA effects for rust resistance.

Incorporating rust resistance, with kernel yield and yield components will enhance groundnut production and productivity. Groundnut breeding should target multiple traits to achieve suitable agronomic performance and high yields. For instance, parental lines Pendo and ICGV-SM 15524 and crosses such as Pendo × ICGV-SM 16589, ICGV-SM 06737 × Pendo and ICGV-SM 15524 × Narinut displayed early flowering and maturity. Hence these genotypes should be selected to improve early maturity for environments with short and erratic rainfall patterns in Tanzania. Chaudhari et al.^48^ and Sukruth et al.^49^ selected groundnut genotypes that exhibited early flowering for yield improvement under marginal conditions especially drought prone areas. Parental lines and crosses that exhibited desirable mean performance in other desirable traits such as higher shelling percent (ICG 12725, ICGV-SM 15524, ICGV-SM 16601, Pendo × ICGV-SM 15524 and ICGV-SM 15567 × ICGV-SM 15524) and hundred seed weight (ICG 12725, ICG 12725 × ICGV-SM 06737, Pendo × Narinut and ICGV-SM 16589 × ICGV-SM 15557) should be selected for direct or indirect selection of grain yield. It is, thus, imperative to consider the correlations existing among the target traits to ensure that appropriate selection methods are devised for simultaneous improvement.

### Combining ability

Except NPP and SP, all assessed traits exhibited significant GCA and SCA variance except NPP and SP, suggesting that the assessed traits were conditioned by additive and non-additive genes. For NPP, the SCA effect was only significant indicating non-additive genes controlling this trait. In addition, the GCA and SCA variances were consistent across seasons indicating that allele interactions and additive gene effects were less influenced by the environment and were thus highly heritable. The traits that exhibit significant GCA effects may be improved by selection and crossing of parental lines with favourable performance for that trait. Parents will be expected to additively contribute their favourable alleles to develop better performing offspring. In contrast, SCA effects will be exploited through hybrid breeding instead of pure line selection. Dominance genes occur as a result of interaction between alleles governing the inheritance of a trait. Intra-allelic interaction is not easily predictable. For instance, two different parents with favourable mean performance for a trait may produce an off springs with undesirable performance due to poor gene combinations or intra-allelic interaction (dominance) or inter-allelic interaction (epistasis). On the other hand, genes from poor performing parents may combine favourably well to produce high performing offspring due to favourable SCA effects. Traits such as rust and leaf spot resistance and grain yield have been reported to be controlled by additive and non-additive gene effects^16,50^. However, other reports indicated that non-additive gene effects were more important for rust resistance and grain yield^51,52^. The significant reciprocal effects in the cross ICGV-SM 16589 × ICGV-SM 06737 (for DTF and DTM) and Narinut × ICGV-SM 06737 for HSW suggested the presence of maternal inheritance effect conditioning trait heritability. Maternal effects are contributed by the female parent and thus it is important to purposefully designate the female and male parents during crosses to exploit any favourable maternally inherited trait. Pasupuleti et al.^53^ and Dwivedi et al.^54^ reported highly significant reciprocal effects for late leaf spot resistance and kernel yield in groundnut, respectively.

### General combining ability of parents

General combining ability analysis is an effective method in selection of parents based on performance of their progenies, usually at the F_I_ or F_2_ and later generations^55^. Developing groundnut cultivars with rust resistance that are adapted to harsh growing conditions is important for sub-Sahara African region where rust and other foliar diseases are endemic. The development of high-yielding cultivar for foliar disease resistance is one of the major objectives of the groundnut improvement programme in Tanzania. Developing suitably adapted cultivars is preceded by identifying parental lines with good combining ability for the suitable traits. Parental genotypes that exhibit good GCA effects often have the ability to transfer their favourable characteristics to the offspring^56^. Parental genotypes such as ICGV-SM 05570 and ICGV-SM 15567 had negative GCA effects for rust resistance, while genotype Kanyomwa had positive GCA effects for grain yield. These parents are selected for developing breeding populations. The GCA effects of the parental lines is particularly important for traits controlled by additive traits since their inheritance and expression in the offspring are conditioned by the summation of the allelic effects of the different parents. For improving traits such as earliness to flowering and maturity, parental lines ICGV-SM 15524 and ICGV-SM 16601 that exhibited negative GCA effect will be ideal due to their potential to reduce the average DTF and DTM in the offspring. Early flowering and maturity varieties are ideal for marginal environments such as those mostly found in sub-Sahara Africa region, characterised by inadequate rainfall and high temperatures. However, earliness to maturity can lead to yield penalty in environments where soil moisture is adequate, and the rainy season is long^57^. Groundnut rust disease epidemiology is favored by continuous warm temperatures ranging between 20 and 30 °C and high humidity above 78%^32,58^. Under favourable moisture and temperature conditions the following genotypes are recommended for breeding: ICGV-SM 05570, ICGV-SM 15559 and Narinut. These lines had positive GCA effects for DTF and DTM useful for long duration variety development. The parents with positive GCA effects for PH (ICGV-SM 15524 and Pendo), NPP (ICGV-SM 15524 and Pendo), HSW (ICGV-SM 15524 and Pendo) and SP (Kanyomwa) will be useful for trait improvement including grain yield. Groundnut genotypes of the Virginia botanical group have high above ground biomass, high number of pods per plant and large seed types which may likely provide higher grain yield. These group of genotypes exhibited medium to late maturity compared to small-biomass types^59^. However, there were no parental lines that exhibited good GCA for all assessed traits in the present study. Therefore, different complementary parents should be selected for breeding purposes based on their GCA effects.

### Specific combining ability of crosses

Specific combining ability effect relates to performance of some crosses relatively better or worse than would be expected based on the average performance of the parents involved^37^. SCA effects represent the non-additive proportion of variance that is difficult to exploit in trait improvement in self-pollinating crops due to low heritability and unpredictability of reshuffling of genes. The performance of a specific cross depends on the extent of the favourable genes for a trait from the two parents complementing each other^55^. For instance, crosses such as ICGV-SM 15557 × ICGV-SM 15559, ICGV-SM 16589 × Narinut and ICG 12725 × ICGV-SM 06737 exhibited good SCA effects for rust resistance and reduced number of days to flowering. The families such as ICGV-SM 06737 × ICGV-SM 15524 and Kanyomwa × ICGV-SM 05570 had good SCA effects for kernel yield due to favourable interaction between alleles from the female and male parents. In some cases, `crosses can exhibit good SCA effect even when their parents have poor or unfavourable GCA effects for the trait. This is due to the favourable interaction after recombination showing that there will be potential for selection of transgressive individuals in segregating populations. Crosses such as Narinut × ICGV-SM 05570 had positive SCA effects and high mean values for kernel yield. Conversely, crosses such as ICGV-SM 16589 × Narinut and ICG 12725 × ICGV-SM 06737 had negative SCA effect and lower mean values for rust severity scores compared to their mid parent values. These suggest that the new progeny are transgressive segregants that could be further selected for trait improvement.

### Gene action

The ratios of GCA to SCA mean squares for most assessed traits were less than one and had a value close to zero based on Baker’s ratio indicating that non-additive gene action had a more prominent role in the control of groundnut rust resistance and agronomic traits. The relatively lower ratios showed the preponderance of non-additive gene effects for most traits and suggested that trait improvement will only be effective after selection in the advanced generations. The non-additive gene action found in this study were in concurrence with^51^, who reported that rust resistance is controlled by non-additive gene action. The significant differences showed by the reciprocal effects indicate that maternal effects have impact on groundnut rust resistance. Hence, it is important to use appropriate mating design like full diallel that will allow to exploit cytoplasmic inheritance. According to^10^ rust resistance is controlled by few genes with either monogenic, digenic or trigenic inheritance and hence backcross breeding can facilitate accumulation of major genes in progenies.

## Conclusion

The assessed groundnut genotypes exhibited wide genetic variation for rust resistance, agronomic traits and kernel yield useful for breeding. The inheritance of rust resistance is conditioned by dominance gene action, while kernel yield was controlled by additive gene action. Parental lines ICGV-SM 05570 and ICGV-SM 15567, which were the best combiners for rust resistance and kernel yield, were selected for breeding population development and pure line maintenance. The individuals ICGV-SM 16589 × Narinut and ICGV-SM 15557 × ICGV-SM 15559 were identified as best specific combiners for rust resistance. The selected families are recommended for genetic advancement and to identify transgressive segregants and develop pure lines for cultivar release and deployment in Tanzania.
